# *Fmr1* Transcript Isoforms: Association with Polyribosomes; Regional and Developmental Expression in Mouse Brain

**DOI:** 10.1371/journal.pone.0058296

**Published:** 2013-03-07

**Authors:** David M. Brackett, Feng Qing, Paul S. Amieux, Drew L. Sellers, Philip J. Horner, David R. Morris

**Affiliations:** 1 Department of Biochemistry, University of Washington, Seattle, Washington, United States of America; 2 Department of Pharmacology; University of Washington, Seattle, Washington, United States of America; 3 Department of Neurological Surgery, University of Washington, Seattle, Washington, United States of America; Duke University Medical Center, United States of America

## Abstract

The primary transcript of the mammalian Fragile X Mental Retardation-1 gene (*Fmr1*), like many transcripts in the central nervous system, is alternatively spliced to yield mRNAs encoding multiple proteins, which can possess quite different biochemical properties. Despite the fact that the relative levels of the 12 *Fmr1* transcript isoforms examined here vary by as much as two orders of magnitude amongst themselves in both adult and embryonic mouse brain, all are associated with polyribosomes, consistent with translation into the corresponding isoforms of the protein product, FMRP (Fragile X Mental Retardation Protein). Employing the RiboTag methodology developed in our laboratory, the relative proportions of the 7 most abundant transcript isoforms were measured specifically in neurons and found to be similar to those identified in whole brain. Measurements of isoform profiles across 11 regions of adult brain yielded similar distributions, with the exceptions of the hippocampus and the olfactory bulb. These two regions differ from most of the brain in relative amounts of transcripts encoding an alternate form of one of the KH RNA binding domains. A possible relationship between patterns of expression in the hippocampus and olfactory bulb and the presence of neuroblasts in these two regions is suggested by the isoform patterns in early embryonic brain and in cultured neural progenitor cells. These results demonstrate that the relative levels of the *Fmr1* isoforms are modulated according to developmental stage, highlighting the complex ramifications of losing all the protein isoforms in individuals with Fragile X Syndrome. It should also be noted that, of the eight most prominent FMRP isoforms (1–3, 6–9 and 12) in mouse, only two have the major site of phosphorylation at Ser-499, which is thought to be involved in some of the regulatory interactions of this protein.

## Introduction

**Figure 1:**
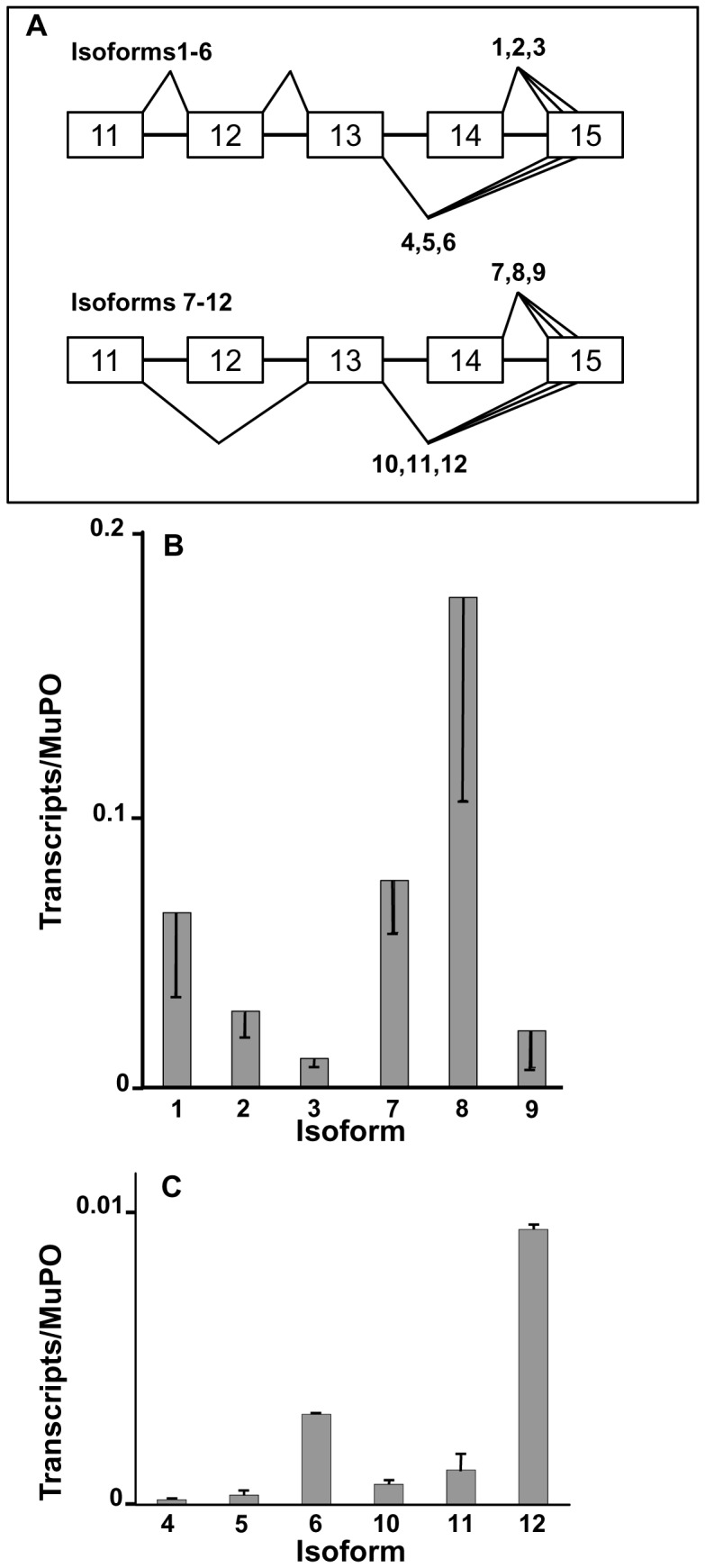
Levels of 12 *Fmr1* transcript isoforms in adult C57/BL6 wild-type whole brain. Total RNA was isolated from each brain and a portion was reverse transcribed using a gene specific primer for *Fmr1* that is present in all splice variants and a MuP0 gene-specific primer. All values are normalized to MuP0 transcript from each brain (Materials and Methods). [**A**] Schematic representation of *Fmr1* alternative splicing patterns in mouse. [**B**] Quantification of transcript levels for higher abundance isoforms. Error bars represent standard deviations for biological triplicates. [**C**] Quantification of transcript levels for lower abundance isoforms. Error bars represent standard deviations of values of triplicate measurements of a single brain. Please see Table 1 for numerical data.

The use of alternative splicing to create repertoires of transcript, and consequently protein, isoforms from single genes is widespread in the mammalian central nervous system, where estimates of 50% to 75% of expressed genes are alternatively spliced [1], [2]. Many reports suggest that alternative splicing of key genes play functional roles in ion channel activity [3], synaptic plasticity [4], the genesis and strengthening of dendritic spines [5], [6], and the structures of neurotransmitter receptors [7]–[9]. The human Fragile X Mental Retardation-1 gene (*FMR1*) is an example of a gene that has the potential to produce a variety of structurally related, yet functionally diverse, protein isoforms through alternative splicing of the primary transcript [10]. It has been suggested that the pre-mRNA (unspliced messenger RNA) transcribed from *FMR1* can be alternatively spliced into as many as 20 different mature transcript isoforms [11]. The longest human *FMR1* mRNA, which shares 97% sequence identity with the mouse *Fmr1* ortholog at the amino acid level (Isoform 1, Fig. 1A), encodes a complex protein of 71 kDa (kilodaltons) that contains a variety of functional sequences and domains, many of which are influenced by alternative splicing of the pre-mRNA [12]–[14]. These putative FMRP (Fragile X Mental Retardation Protein ) isoforms 1) may bind with different affinities to RNA structures [15], 2) show the presence or absence of a nuclear export sequence, and 3) possess altered regions of post-translational modification [12], [13].

The effects of alternative splicing on the protein products of the *Fmr1* gene are potentially quite remarkable, producing a family of FMRP isoforms with subtle and not so subtle differences that are predicted to profoundly affect the biochemical functions of the proteins. FMRP Isoform 1 is the full-length protein, which contains at least three RNA-binding domains (two K-homology domains and an RGG box) [16], nuclear export and localization signals [12], [17], and sites for post-translational modification through phosphorylation and methylation [12], [18]. Use of the first alternative splice acceptor site in Exon 15 (see Fig. 1), producing FMRP Isoform 2, deletes the key phosphorylation site (Ser-499 in mouse). Since protein Isoform 2 would be insensitive to the class of signal transduction pathways that target this site, significant biological implications are implied; FMRP phosphorylation has been suggested to be involved in translational control in neurons [18]. Use of the second alternative acceptor site in Exon 15 (Isoform 3, Fig. 1) maintains the deletion of Ser-499 and also removes amino acids that comprise the recognition site required for methylation of the protein [13], possibly through a conformational switch [19]. This same splicing pattern with respect to Exon 15 is mirrored in Isoforms 7–9, however, these isoforms are also missing Exon 12 (Fig. 1), which has the effect of shortening a loop between the β2 and β’ strands within the second KH (K-homology domain ) domain of the protein [20]. The KH domain of Isoforms 7–12, which has the truncated “variable loop”, has been shown to interact with a complex tertiary RNA fold termed a “kissing complex” that mediates association of certain mRNAs with FMRP Isoform 7 [21]. The extended variable loop of Isoforms 1–6 forms part of the putative RNA-binding cleft of the KH2 domain [22]. The influence of this variable loop on the specificity of cargo binding in neurons is not known, although the presence or absence of this loop may have subtle effects on binding to a loop-loop pseudoknot ligand, although interpretation of these results are influenced by apparent differences in stability of the protein isoforms [23], [24]. FMRP Isoforms 8 and 9 are identical to Isoform 7 in the region of the second KH domain [21] and they have the modified signal-transduction properties predicted for Isoforms 2 and 3. Skipping of Exon 14, with formation of Isoforms 4–6 and 10–12 (Fig. 1), has no influence on the KH-domains of FMRP, but deletes the nuclear export signal encoded by Exon 14. Exclusion of Exon 14 also results in a +1 frame shift in the coding sequence of the *Fmr1* transcripts, producing novel C-termini for these proteins and loss of sites of post-translational modification and the RGG box. The RGG box is an RNA-binding domain that binds RNA molecules containing the “G-quartet” structure [25]. It is of note that loss of the nuclear export sequence retains these six isoforms of FMRP in the nucleus [12], which is likely of functional significance.

The biochemically dissimilar isoforms of FMRP could occur together in the same cell type or separately in distinct cell types. Although at least 4–5 of the predicted protein isoforms have been identified in mouse brain by Western blot analysis [26], [27], a systematic study of the occurrence of the *Fmr1* transcript isoforms in the central nervous system and their translation into protein has yet to be reported. A complete analysis of FMRP isoforms in human or mouse tissue has not been possible due in part to a lack of isoform-specific antibodies. To address the issue of FMRP isoform distribution, we document here a comprehensive analysis of the expression of 12 *Fmr1* transcript isoforms in adult mouse brain, as well as during embryonic and postnatal development. Although the relative levels of the transcript isoforms differ by as much as two orders of magnitude, all are associated with polyribosomes of a size consistent with translation into the corresponding FMRP isoforms. The isoform compositions of regions of adult brain that display continuing neurogenesis show patterns similar to those observed at early stages of development and also in cultured neural progenitor cells.

## Results

### Levels of *Fmr1* Transcript Isoforms in Adult Mouse Brain

Twelve *Fmr1* transcript isoforms were measured in whole brains of wild-type C57BL/6 mice by real-time reverse transcriptase PCR (qRT-PCR). The fidelity of the primers employed for amplification of individual isoforms was established by mobility of the amplified products in agarose gels and sequence analysis (see Materials and Methods). The values of the transcript levels in each tissue were normalized to the level of the transcript encoding the mouse ortholog of the housekeeping gene human acidic ribosomal protein (MuP0), also determined by qRT-PCR, providing molar ratios of the transcripts (see Materials and Methods). The human ortholog of MuP0 has been shown to be a reliable housekeeping gene for normalization [28], [29], and MuP0 is uniformly expressed across mouse brain regions (P. Amieux, unpublished results).

As can be seen from Fig. 1 (B and C), all 12 transcript isoforms are expressed at significant, but widely varying levels in whole brain. In general, skipping of Exon 12 (Isoforms 7–12) is a more frequent event than its inclusion (Isoforms 1–6; see Fig. 1A for the structures of the transcripts), a result consistent with previous reports in both mouse and human brain [30]. Of the isoforms containing Exon 12, Isoform 1 is the most abundant, but it is nevertheless 2- to 3-fold less abundant in adult brain than Isoform 8. The six *Fmr1* mRNAs from which Exon 14 has been excluded (Isoforms 4–6 and 10–12, Fig. 1C) are expressed at very low levels, ranging from 7- to nearly 1000-fold lower abundance than Isoform 8 (Fig. 1B). Exclusion of Exon 14 from the *Fmr1* transcripts results in a more frequent use of the second alternative acceptor site in Exon 15, yielding a bias towards Isoforms 6 and 12, a result also consistent with the literature [12], [30]. The numerical values for these data are presented in Table 1.

**Table 1:** Numerical values for transcript isoform levels in adult mouse brain^a^.

More abundant isoforms			Less abundant isoforms		
Isoform	Level^b^	Error^c^	Isoform	Level^b^	Error^c^
1	0.062	±0.030	4	0.00014	±8.3E−05
2	0.027	±0.010	5	0.00031	±1.5 E−04
3	0.009	±0.003	6	0.00300	±4.1E−05
7	0.074	±0.019	10	0.00060	±1.4E−04
8	0.176	±0.074	11	0.00110	±5.0E−04
9	0.019	±0.014	12	0.00936	±2.1E−04

a These are the numerical values for the experiment results shown in Fig. 1.

b Molar ratio of isoform/MuP0 transcripts (Fig. 1).

c Standard deviation of replica measurements.

### Association of *Fmr1* Transcripts with Polyribosomes

**Figure 2:**
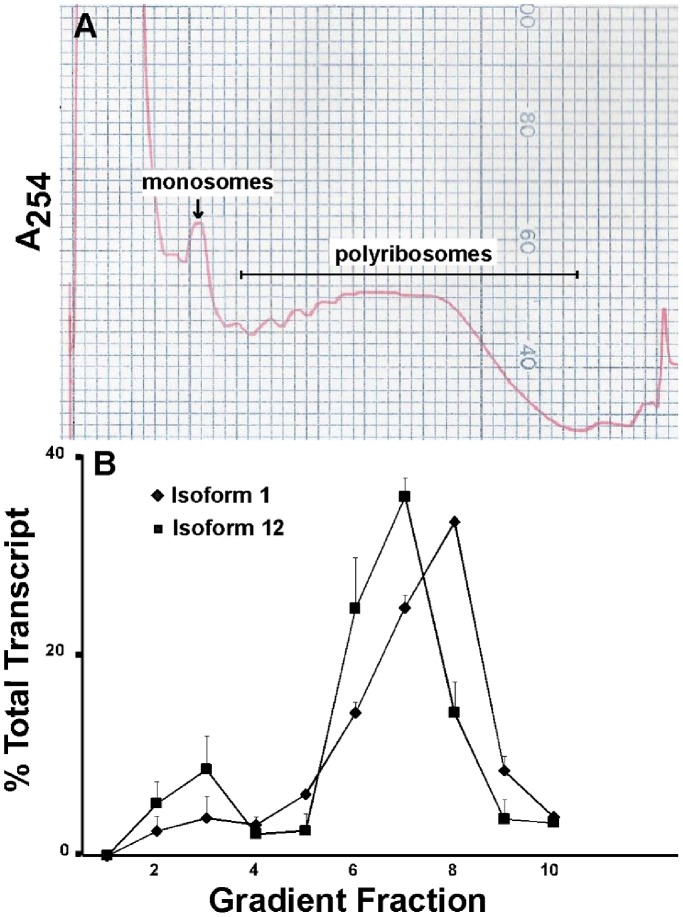
Polyribosome profiles of the longest and shortest *Fmr1* transcript isoforms. An adult mouse was euthanized by CO_2_ asphyxiation and decapitated. The brain was quickly removed and placed into liquid nitrogen. The brain was then later homogenized (Materials and Methods) and a portion was loaded onto a linear 15–60% sucrose gradient. Fractions were collected by upward displacement. [**A**] A_254_ profiles were determined using an ISCO fractionator. [**B**] Carrier *E.coli* RNA and UT2 internal standard mRNA was added to each fraction prior to RNA extraction. The amount of RNA lost during extraction was determined by qRT-PCR of the UT2 mRNA (Materials and Methods). *Fmr1* isoforms 1 and 12, the longest and shortest forms, were quantified across the sucrose gradient by qRT-PCR. Error bars represent variation in replicate measurements from one brain.

As shown in Fig. 1, all 12 *Fmr1* transcript isoforms are expressed at significant, but quite different levels in adult mouse brain. An important question is whether all of these transcripts, even those at lowest abundance, are associated with actively translating ribosomes or whether some of these splicing variants are simply dead-end products that are not translated into protein. To answer this question, we investigated the degree of association of the individual *Fmr1* mRNA isoforms with polyribosomes. Extracts of whole mouse brain were fractionated by sucrose gradient centrifugation [31]. Absorbance at 254 nm was monitored after centrifugation and the absorbance profile was used to identify the position of single ribosomes and polyribosomes (Fig. 2A). Treatment with low concentrations of RNase collapses the polysome region of the absorbance profile to the region of monosomes sedimenting at 80S (see Supporting Information, Fig. S1). Fractions were collected across the gradient and a commercially available control mRNA (UT2) was added to each fraction prior to RNA extraction to monitor recovery (see Materials and Methods). The profiles of *Fmr1* transcript isoforms were monitored by qRT-PCR. The results for two isoforms, one abundant (Isoform 1, longest transcript) and one rare (Isoform 12, shortest transcript), are presented in Fig. 2B. Greater than 80% of both transcripts sediments in a region of the gradient consistent with association with multiple ribosomes. Also consistent with ribosomal association, chelation of magnesium ion in the extracts with EDTA relocated 90–100% of the *Fmr1* transcripts towards the top of the sucrose gradient (see Supporting Information, Figs. S2 and S3). The peak of Isoform 1 mRNA is located in fraction 8, which based on the A_254_ profile corresponds to polyribosomes containing approximately 11 ribosomes per transcript. Analysis of the same sucrose gradient fractions revealed the peak of the Isoform 12 transcript to be in fraction 7, corresponding to 9 ribosomes loaded on the transcripts in this region of the gradient. This difference in sedimentation is predicted from the shorter open reading frame in transcript Isoform 12 (see Discussion). The results in Fig. 2B are typical of all 12 transcript isoforms (summarized in Supporting Information, Figs. S2 and S3), which range between 55 and 90% contained in the polyribosome fractions.

### Pattern of *Fmr1* Transcript Isoforms Expressed in Neurons

**Figure 3:**
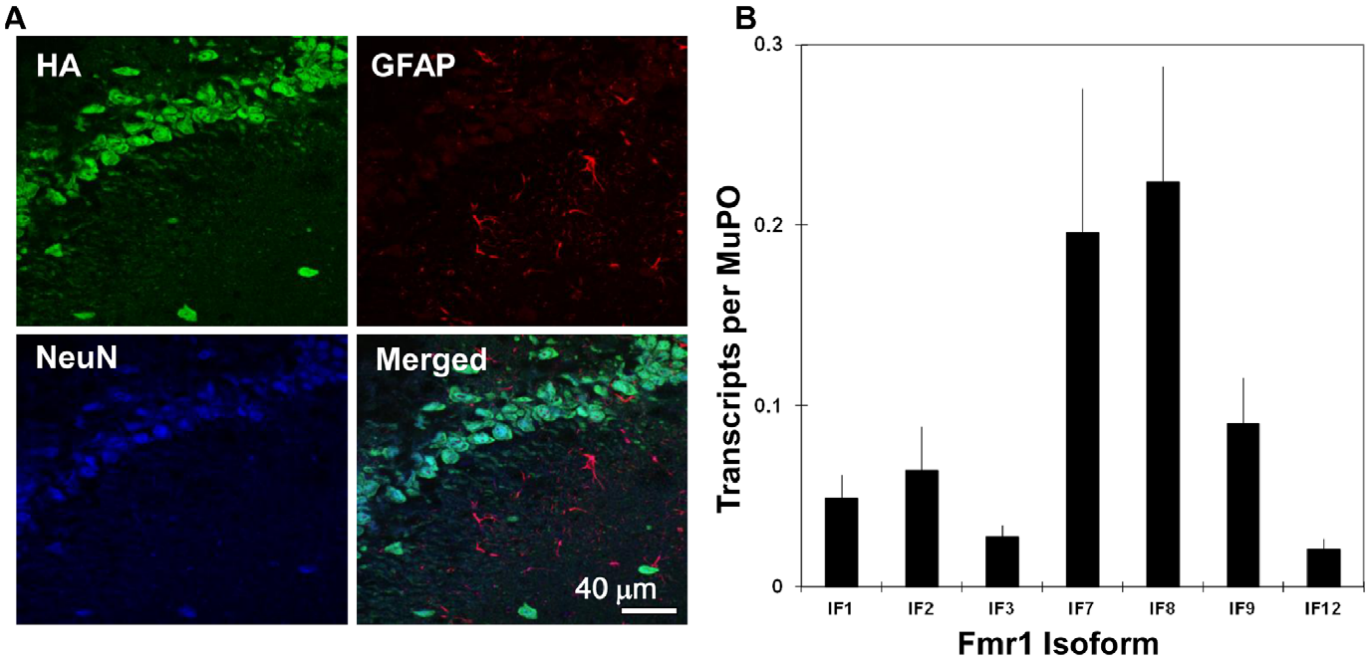
*Fmr1* transcript isoforms enriched on neuron-specific ribosomes from adult mouse brain. The neuron-specific, ribosome-bound transcripts were enriched using the RiboTag methodology as described in the text. (**A**) Immunohistochemistry on paraformaldehyde fixed coronal brain sections from Eno2-Cre:RiboTag mice demonstrating neural specific expression of Rpl22-HA (HA staining); sections are stained with the neuronal marker NeuN and counterstained with the glial specific marker GFAP. Overlap of NeuN and HA staining and absence of overlapping HA and GFAP staining supports neuron-specific labeling of ribosomes. This particular section corresponds to the dentate gyrus area of the hippocampus. Immunohistochemistry in cortex, striatum and other brain regions was similar to the image shown, suggesting that HA staining is specific to neurons. Scale Bar = 40 microns. (**B**) The Fmr1 transcript isoforms were isolated from immuno-purified polyribosomes and quantified by qRT-PCR as in Fig. 1. The results are averaged values from five independent, neuron-tagged mice. The average degree of neuronal enrichment of the tagged ribosomes over these five experiments was 3.2±0.7 fold, as estimated using the neuron-specific transcript from the *NeuN* gene (Fig. S4).

The patterns of transcript isoforms described above are derived from whole tissue samples and reflect expression in all cells of the brain. Recent reports indicate that 35–50% of the cells in mammalian brain are non-neuronal in nature [32]–[34]. FMRP is expressed in cells of the astrocyte lineage during development [35] and astrocyte function is affected in cells lacking the protein [36]–[38]. We have developed the RiboTag mouse, which employs cre-lox methodology to express epitope-tagged ribosomes in specific cell types [39]. The RiboTag approach allows access to translated transcriptomes in specific cell types of interest. For this study, we performed a cross between the RiboTag mouse and a mouse expressing the neuron-specific *Eno2*-cre [40]. This resulted in tagging the ribosomes with HA specifically in neurons, with no label in glia (Fig. 3A). Immunoprecipitation of polyribosomes from brain extracts of the progeny from this cross provided neuron-specific transcriptomes for analysis of the Fmr1 transcript isoforms. The efficacy of the immunoprecipitation was shown by a 3.2±0.7 fold enrichment of the neuron-specific NeuN transcript and a 0.56±0.12 fold depletion of the glia marker GFAP (Fig. S4). The relative levels of the most abundant Fmr1 isoforms, averaged from five independent mice, are provided in Fig. 3B. Inspection of these results obtained from the neuron-enriched, translated transcriptomes reveals no significant differences in isoform patterns compared with whole brain (Fig. 1). Therefore, since non-neuronal cells comprise nearly 50% of the cells in brain, if *Fmr1* is expressed in these cells, the pattern of splicing of the major isoforms must be indistinguishable from that in neurons.

### *Fmr1* Transcript Isoforms in Developing Mouse Brain

**Figure 4:**
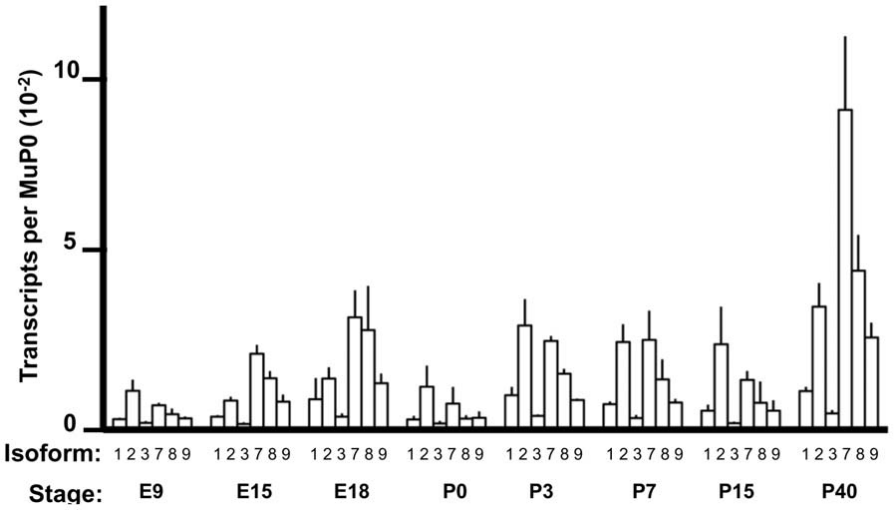
Expression of the major *Fmr1* isoforms during mouse brain development. Brains were harvested in triplicate from male littermates and *Fmr1* transcript isoforms were quantified as described in Materials and Methods. The data for all time points (except P7) are averages of biological triplicates and error bars represent the standard deviation in the biological triplicates. The values for P7 are an average of two brains, and the error bars represent the range in data.

Previous reports have shown *Fmr1* expression in human and mouse embryonic brains [41], [42]. To explore the *Fmr1* transcript isoforms during pre- and postnatal development, whole brain was dissected on ice from male wild-type C57BL/6 littermates (Materials and Methods) at stages ranging from embryonic day 9 (E9) to postnatal day 40 (P40). Expression of *Fmr1* transcript isoforms was quantified in these samples and a full developmental series for the most abundant isoforms is shown in Fig. 4.

**Figure 5:**
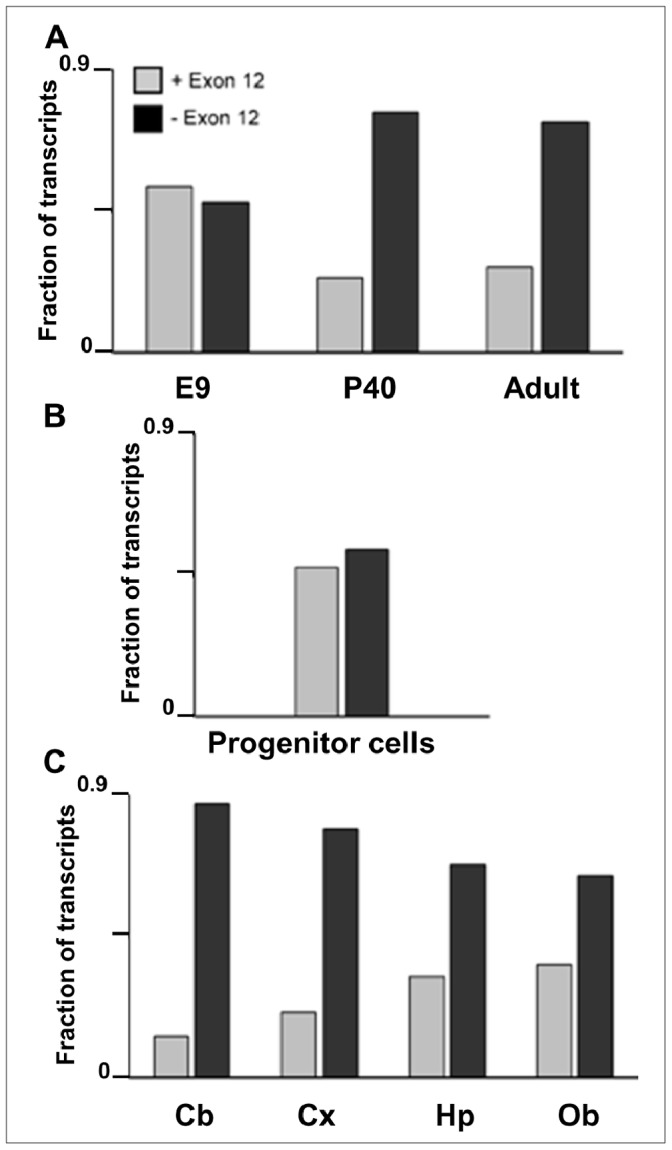
Exon 12 in *Fmr1* transcripts from mouse brain and cultured progenitor cells. The levels of the abundant *Fmr1* transcripts were measured in [**A**] embryonic day 9 (E9), postnatal day 40 (P40) and adult mouse brains, in [**B**] cultured neural stem/progenitor cells, and in [**C**] dissected regions of adult mouse brain [cerebellum (Cb), cortex (Cx), hippocampus (Hip), and olfactory bulb (Ob)]. The data are presented as the fractions of *Fmr1* transcripts containing (gray bars) and lacking (black bars) Exon 12 (eg. “+ Exon 12″ is the sum of Isoforms 1–3 divided by the sum of Isoforms 1–3 plus 7–9). Error bars for the individual isoforms are found in Fig. 1 for adult whole brain, in Fig. 4 for E9 and P40, in Fig. 6 for cultured neural stem/progenitor cells, and in Figs. 7 and 8 for individual brain sections.

One of the most notable changes in isoform distribution during neural development involved the presence or absence of Exon 12 (Isoforms 1–6 versus 7–12). These two families of isoforms are defined by the size of the variable loop in the KH2 RNA-binding domain of the protein (please see Introduction). The results for E9, P40 and mature adults are summarized in Fig. 5A. At the early embryonic point (E9), the two families of isoforms are present at approximately equal levels. The relative levels of the two isoform families change during development, so that by P40 the isoforms lacking the extended loop in the KH2 domain out-number Isoforms 1–6 by three to one, which is similar to the ratio in mature adults (Fig. 5A). These results suggest a possible shift in RNA-binding properties of FMRP during development (see Discussion).

### *Fmr1* Transcript Isoforms in Cultured Neural Progenitor Cells

**Figure 6:**
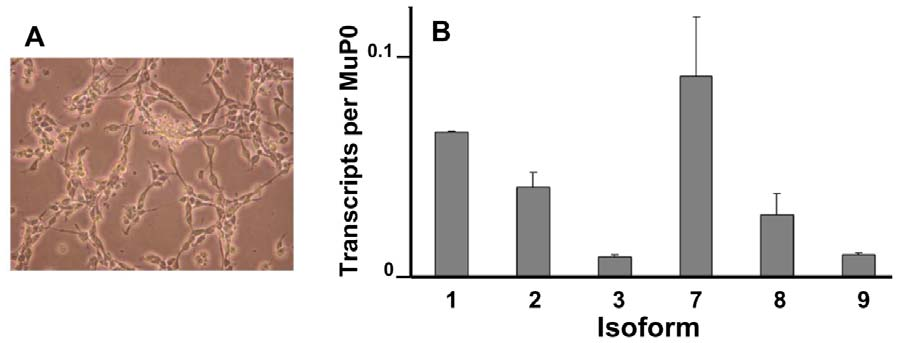
The abundant *Fmr1* transcript isoforms in cultured neural stem/progenitor cells . Neural stem/progenitor cells were maintained as described in Materials and Methods. An unstained phase contrast micrograph of the cultured cells is shown in [**A**]**.** Levels of the most abundant transcript isoforms were determined in triplicate biological samples and the error bars represent standard deviations of the means [**B**].

The shift in structure of the KH2 domain during development suggested that the isoform composition of neural progenitor cells might resemble that of early embryonic brain. To test this hypothesis, *Fmr1* expression was measured in undifferentiated neural stem/progenitor cells in culture (Fig. 6A). In undifferentiated neurospheres, the relative levels of Isoforms 1–6 and 7–12 were very similar (Fig. 5B), approximating the results from E9 embryonic brains (Fig. 5A). The values of the abundant *Fmr1* transcript isoforms in neural stem/progenitor cells are documented in Fig. 6B.

### *Fmr1* Expression in Anatomical Regions of Mouse Brain

**Figure 7:**
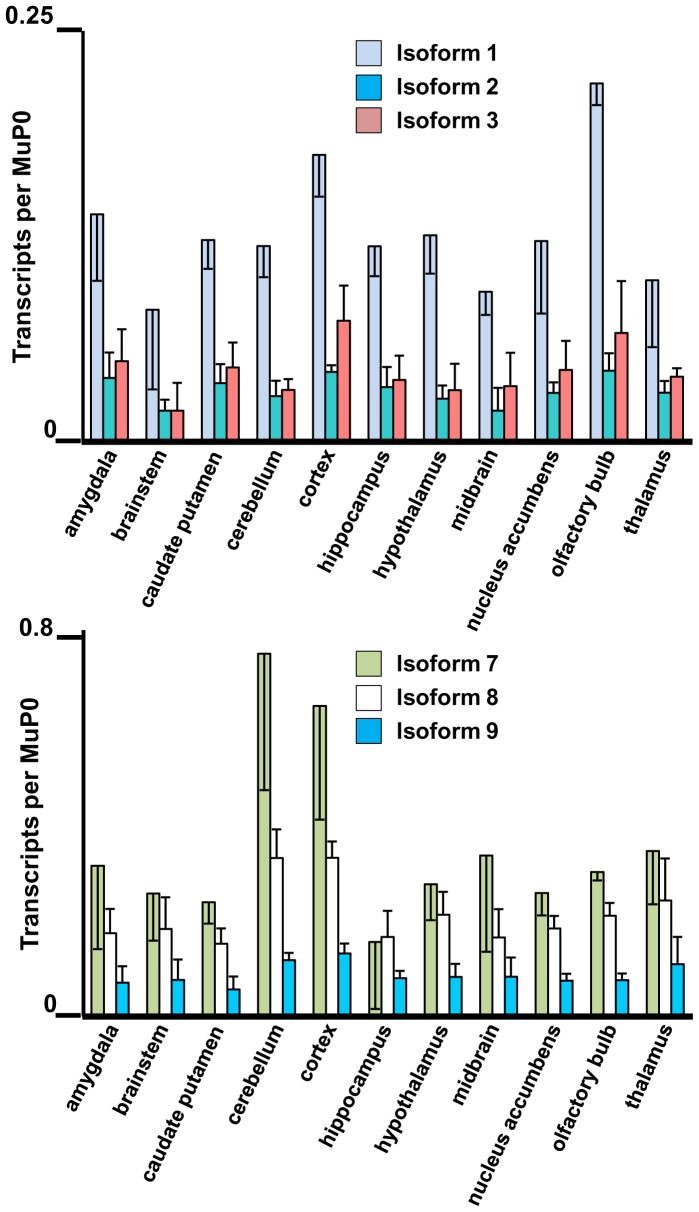
Highly expressed *Fmr1* transcript isoforms in eleven brain regions. The amygdala, brainstem, caudate putamen, cerebellum, cortex, hippocampus, hypothalamus, midbrain, nucleus accumbens, olfactory bulb and thalamus were dissected from three adult mouse brains. The levels of the abundant Fmr1 transcripts are shown with standard deviations in biological triplicates.

**Figure 8:**
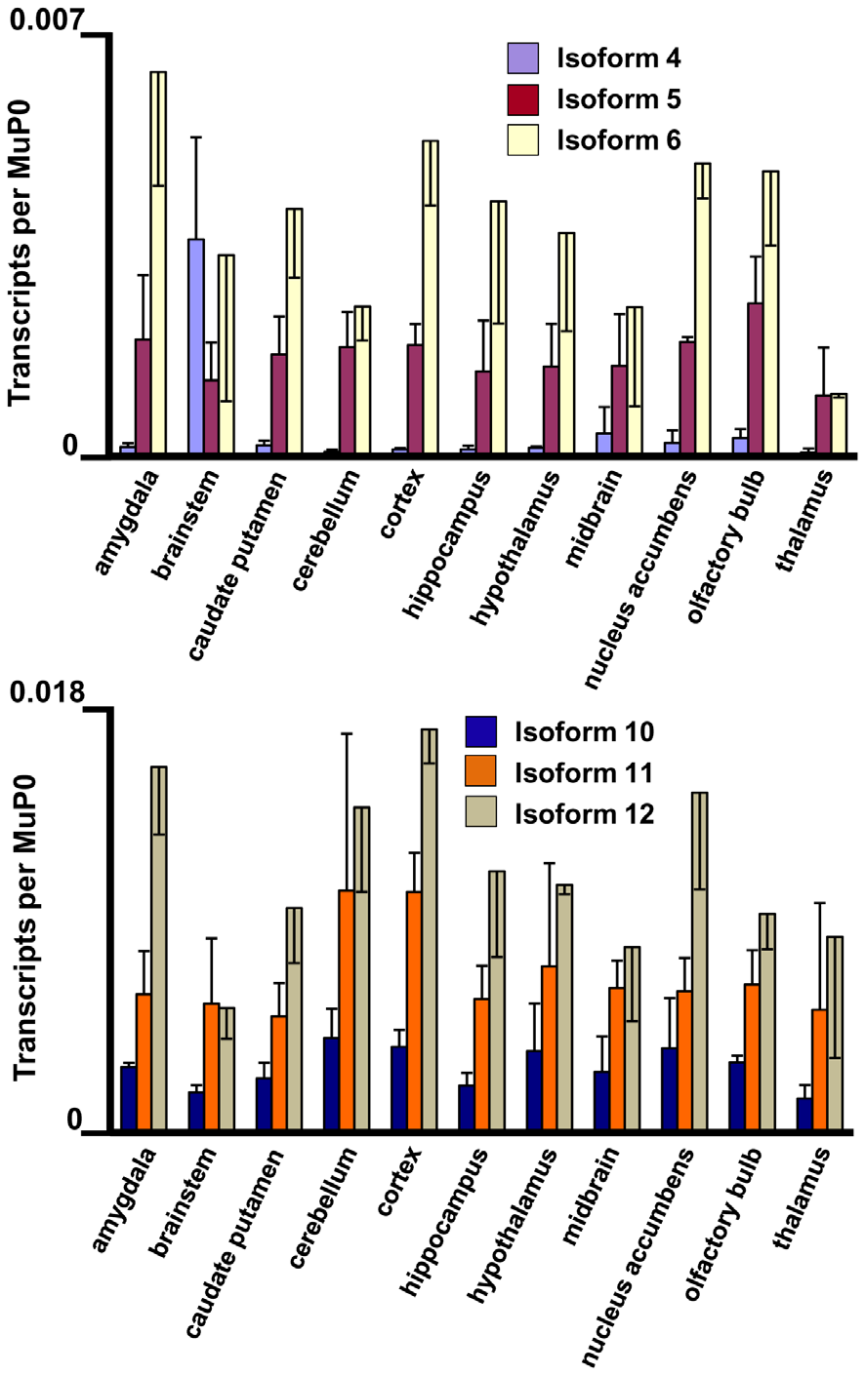
Low expressed *Fmr1* transcript isoforms in eleven brain regions. Data for isoforms 4–6 and 10–12 are as indicated. The brain regions are defined in the legend to Fig. 7.

Eleven anatomical regions were dissected from adult mouse brain and total RNA was extracted for quantification of *Fmr1* isoforms. All 12 *Fmr1* transcript isoforms are expressed at a significant level in the eleven regions (Figs. 7 and 8). The relative levels of Isoforms 1–6 (contain Exon 12) and 7–12 (lack Exon 12) in four prominent brain regions are presented in Fig. 5C. All four regions show the bias towards Isoforms 7–12 that is characteristic of adult brain. However, in the hippocampus and olfactory bulb the levels of Isoforms 1–6 relative to MuP0 are approximately double those in the cortex and cerebellum. One common feature of the hippocampus and olfactory bulb is that they share the property of significant neurogenesis in the adult. The ratios of the *Fmr1* isoforms with the variant KH2 domains in these tissues, together with the results in developing mouse brain and neural progenitor cells, suggest that possible differences in RNA-binding properties of the proteins encoded by these transcripts may be important for neurogenesis (see Discussion).

## Discussion

Real-time RT-PCR is a sensitive and efficient method for quantification of single RNA species in any tissue type. However, the strengths of the method can be compromised when comparing levels of different transcripts, since small differences in amplification efficiencies can lead to large errors in comparisons using methods such as ΔC_t_ (threshold cycle) [43], [44]. In the current study, in order to compare the absolute levels of the *Fmr1* transcript isoforms, we employed a novel approach that used the isolated amplification products (amplicons) generated by each primer set to produce standard curves. This strategy resulted in absolute quantification of the level of each isoform, independent of the individual amplification efficiencies. By applying this approach to both the *Fmr1* isoforms and the housekeeping gene MuP0 used for normalization, it was possible to reliably compare the levels of the individual isoforms between themselves and between different tissues.

Multiple species of FMRP have been detected previously using Western blots [26], [27], consistent with at least some of the predicted protein isoforms being synthesized. However, because of similarities in the molecular weights of many of the putative FMRP isoforms, it was impossible to explore by Western blots the full extent to which the transcript isoforms were translated. The studies presented here argue that all twelve of the transcript isoforms examined are associated with polyribosomes and therefore are likely translated into the corresponding protein isoforms. Association of the *Fmr1* transcript isoforms with translating ribosomes is suggested not only by the important control experiment showing sensitivity to EDTA, but also by the relative positions of the transcripts in the sucrose gradients. As noted in presenting the results of Fig. 2, the Isoform 1 transcript sediments at a position consistent with association with an average of 11 ribosomes. Given the length of the open reading frame of Isoform 1 (1842 bases), this transcript contains a calculated density of one ribosome per 167 bases. This value compares well with the average ribosome density on other eukaryotic transcriptomes [45]. Isoform 12 has the shortest open reading frame of the *Fmr1* transcripts (1473 bases) and its apparent association with an average of 9 ribosomes yields a density of one ribosome per 163 bases. The most straight-forward interpretation of these results is that the two most structurally divergent *Fmr1* transcripts carry the same density of ribosomes and therefore are translated into protein with equal efficiencies in brain. It would be difficult to reconcile these results with the sedimentation rates of these transcripts being dictated by association with non-translating particles.

The transcript isoforms lacking exon 14 (Isoforms 4–6 and 10–12) are potentially substrates for nonsense-mediated decay because of a shift in the reading frame introduced as a consequence of the exon omission. If these truncated proteins are produced, their functions are of interest since, unlike the major isoforms, they should be localized solely in the nucleus due to the loss of the nuclear export signal. To our knowledge, these minor protein isoforms have not been directly identified, since Western blots have insufficient resolving power and relevant data from mass spectroscopy are not available. On the other hand, we have isolated and sequenced full-length cDNAs encoding Isoform 6 and Isoform 12 at a frequency of 1 in 16 total *Fmr1* clones (unpublished), demonstrating that the intact transcripts exist at significant levels in brain tissue. Additionally, as noted above the number of ribosomes calculated to be associated with the Isoform 12 transcript would be consistent with it being translated at the same efficiency as the more abundant transcripts. However, direct evidence relating to the existence of the minor FMRP isoforms and their intracellular localization is truly needed.

Striking differences in the biochemical properties of the various FMRP isoforms suggest that variations in absolute expression or in ratios between the isoforms may be of interest biologically. Although it is clearly premature to interpret the current studies in mechanistic detail, in part because of the cellular complexity of the tissues involved, some interesting features of the expression patterns are of note. In cultured neural stem/progenitor cells, the ratios of transcript isoforms encoding the FMRP variants with and without the extra loop sequence in the KH2 domain approximated 1.0 (Fig. 5B), a pattern which was also found in the transcripts from developmental day E9 (Fig. 5A). Interestingly, areas in the adult brain with the highest ratios of these isoforms were the hippocampus and the olfactory bulb (Fig. 5C). This may be reflective of the significant populations of neural stem/progenitor cells in the dentate gyrus region of the adult hippocampus and also the continuous influx of neuroblasts from the subventricular zone into the olfactory bulb [46]–[48]. In contrast, other regions of adult brain showed a stronger bias towards the transcript isoforms lacking Exon 12 (Figs. 5C, 7 and 8). Although the functional role of the loop sequence encoded by Exon 12 in the second KH domain has yet to be defined, it is indeed suggestive that this sequence lies along one side of the putative RNA-binding cleft of the KH2 domain [22]. If the absence of this extended loop were to modify the RNA binding properties of the protein, the observed changes in ratios of the isoforms would alter the spectrum of mRNA molecules bound by FMRP during neurogenesis in both the developing embryo and the adult.

It is of note that, of the eight most prominent isoforms (1–3, 6–9 and 12) of FMRP in mouse, only two have the phosphorylation site at Ser-499. One expects that post-translational modification would significantly affect the biological functions of FMRP. Phosphorylation of this universally conserved serine residue [49] is thought to modulate the influence of FMRP on translation of the mRNAs that it binds [18], [50]–[52]. The “mGluR (metabotropic glutamate receptor ) theory” of Fragile X Syndrome has suggested a role for FMRP in regulating translation in dendritic processes [53]–[56] and implicit in this theory is the ability of the protein to be phosphorylated. It follows that the mGluR theory involves only FMRP Isoforms 1 and 7, while phosphorylation-independent functions should be considered for the remaining isoforms.

The fact that the *Fmr1* transcripts studied here are apparently translated has definite implications with regard to our view of the biology of human Fragile X Syndrome. The physiological and behavioral phenotypes of individuals with this condition clearly result from the net loss of an entire family of proteins, which likely have a variety of different predicted biochemical properties as noted above. Deeper understanding of the physiological roles of individual FMRP family members in intact cells and organisms should be informative in designing treatments for this condition, which is the leading cause of inherited mental retardation in human males.

## Materials and Methods

### Ethics Statement

All animal work was conducted according to national and international guidelines. Protocol 4171-01 was approved by University of Washington IACUC.

### *Fmr1* Transcript Isoform-specific Primers

All primers were designed using the mouse *Fmr1* Isoform 1 cDNA sequence (Gene Accession NM_008031) as a reference, with exon boundaries and naming of isoforms based on previous reports [11], [30]. A schematic and the sequences of the primer sets are given in Fig. S5. In order to quantify half of the *Fmr1* transcript isoforms by qRT-PCR (real-time reverse transcriptase PCR), primer sets for the most abundant transcripts produce amplification products that range between 301 and 347 nucleotides (outside of the recommended 88–200 nucleotide range). The larger size of these amplification products has no detrimental effect on efficiency of the qRT-PCR reactions, with efficiencies for these primer sets ranging from 90–100% (data not shown). Two forward primers ([Iso 1–6L] and [Iso 7–12L]) recognize either the inclusion of Exon 12 (Isoforms 1–6) or the Exon 11–13 junction (Isoforms 7–12). A total of six different reverse primers are used in combination to recognize the use of different splice sites in Exon 15. The [Iso 1, 7R] primer recognizes a region in Exon 15 unique to the full-length Exon 15 and unique to Isoforms 1 and 7; [Iso 2, 8R] primer recognizes the Exon 14 to first alternative acceptor site in Exon 15 (15_1_) junction (14–15_1_); [Iso 3, 9R] primer recognizes the Exon 14 to second alternative acceptor site in Exon 15 (15_2_) junction (14–15_2_); [Iso 4, 10R] primer recognizes the Exon 13–15 junction; [Iso 5, 11R] primer recognizes the 13–15_1_ junction; Iso 6, 12R primer recognizes the Exon 13–15_2_ junction. Only [Iso 1–6L], [1, 7R], and [Iso 3, 9R] have 100% homology to any published *Fmr1* sequence because only Isoform 1 and Isoform 3 intact mRNA sequences have been published. However, all primers are perfect matches to the exon junctions they are designed to identify. We have isolated 5 *Fmr1* cDNAs (Iso 1, 3, 7, 9, and 12, data not shown) and the primers used to identify these sequences have 100% homology. The Exon 13 portion of primer [1–6L] has near homology to the 5′ portion of intron 12, however, unspliced pre-mRNA would not be detected in our qRT-PCR experiments due to the inability of a short 30 second extension spanning 2118 nucleotides of intron 12. The amplification product of all primer sets were run on 1% agarose gels, stained with ethidium bromide, and visualized with UV light (Figs. S6 and S7). The bands were shown to be of the correct nucleotide length and were excised and verified by sequence analysis.

### Animal Tissues

All animals were euthanized by CO_2_ asphyxiation in accordance and with approval of the local University of Washington Institutional Animal Care and Use Committee (IACUC). All brain samples were harvested from C57BL/6 mice at various ages in triplicate, when possible. For the brain region study (Figs. 7 and 8), the brains of 3 adult mice were removed after asphyxiation and sectioned using a Rodent Brain Matrix (Harvard Apparatus). Each section was micro-dissected into brain regions on a titanium metal block on ice. For the developmental series, we used 3 male littermates for each time point. Embryos were euthanized in accordance and with the approval of the University of Washington Institutional Animal Care and Use Committee (IACUC). Sex determination for E9 to P0 mice was performed using PCR primers for the Sry gene (sex-determining region on the Y-chromosome, forward: gcacagagattgaagatcctacac, reverse: gctgctggtggtggtcatag). Older littermates were sexed according to visualization of dissected testes after euthanasia and brain removal.

### Cultured Neural Stem/Progenitor Cells

Cultured neural/stem progenitor cells were maintained in N2 media containing 20 ng/mL FGF (fibroblast growth factor ) and 20 µg/mL EGF (epidermal growth factor), as previously described [57]. A micrograph of the undifferentiated cells is shown in Fig. 6A.

### Reverse Transcription

RNA was extracted from tissue samples and cell lines using RNeasy Mini Kits (Qiagen) and reverse transcription (RT) reactions were done with SuperScript III Reverse Transcriptase (InVitrogen), according to the manufacturer’s recommendations. The reverse transcription reactions of RNA samples, in which *Fmr1* transcript levels were quantified, used gene-specific RT primers for both *Fmr1* and MuP0 (Gene Accession NM007475). The gene-specific RT primers for both *Fmr1* and MuP0 were located approximately 100 nucleotides to the 3′ side of the region used to quantify both genes. The Fmr1 gene-specific primer is located in Exon 16, and is common to all splice variants, (TTCCTTTAGCCTCTCTTGGATTAC). The MuP0 reverse primer is AGGCCTTGACCTTTTCAGTAAG. RNA reverse transcribed from polyribosome gradients used the *Fmr1* gene-specific RT primer and the reverse primer for the ‘spike-in’ control RNA, UT2.

### *Fmr1* Amplicons

Due to the requirement of primer design to identify novel splicing junctions, not all primer sets have maximal or exactly the same amplification efficiency in qRT-PCR reactions. We chose to use an absolute quantification approach that allows us to relate quantitative data for all isoforms to one another, regardless of differences in amplification efficiency. The PCR product for each *Fmr1* isoform primer set (amplicon) was purified on a 1% agarose gel and excised using a gel extraction kit (Qiagen). The amplicons were diluted to 200 fg/µL, 20 fg/µL, 2 fg/µL, 0.2 fg/µL, and 0.02 fg/µL (based on A_260_ measurements of the purified DNA). These amplicon dilutions were then used in qRT-PCR reactions for absolute quantification of each isoform. The purified amplicons were also used to demonstrate that the primer sets are specific, with no cross-reactivity between unrelated amplicons and primer sets. Amplification efficiencies were determined to be equal within a primer set regardless of the source of DNA (amplicons vs. cDNA, data not shown). No data were used in which amplification efficiencies were below 85%. A MuP0 amplicon was prepared as described above, with the following primers for PCR, forward: TGTTTGACAACGGCAGCATTT, reverse: CCGAGGCAACAGTTGGGTA. All RNA, which was reverse-transcribed from brain, used an *Fmr1* gene-specific RT primer and a MuP0 gene-specific RT primer (except RNA from polyribosome fractions, see below). Prior to *Fmr1* quantification, MuP0 was measured in samples by the same absolute quantification method described above. These data were then used to normalize the quantitative data obtained for *Fmr1* from the same RT samples.

### Quantitative RT-PCR

All qRT-PCR reactions were performed on a BioRad iCycler using either POWER SYBR green (ABI) or SensiMix DNA kit (Quantace) master mixes. Cycling conditions for all qRT-PCR reactions were 98°C for 10 min, then 40 cycles of 98°C for 30 s, 60°C for 25 sec, and 72°C for 45 sec, followed by a standard melt curve.

### Polyribosome Analysis

An adult male C57BL/6 mouse was euthanized by CO_2_ asphyxiation in accordance and with the approval of the University of Washington Institutional Animal Care and Use Committee (IACUC). The brain (approximately 500 mg) was subsequently isolated after decapitation and immediately homogenized (10% w/v homogenate) by 10 strokes in a baked, ice-cold Dounce homogenizer with 5 mL lysis buffer (no detergent, no RNase inhibitors) containing 250 mM sucrose, 50 mM Tris, pH 7.5, 100 mM KCl, and 12 mM MgCl_2_ as described [58], [59]. A post-mitochondrial supernatant (S1) was obtained by spinning homogenates at 16,000 g for 10 minutes at 4°C. Approximately 20 A_260_ units of S1 was loaded onto a linear 15–60% sucrose gradient (identical salt and buffering conditions as the homogenization buffer) followed by centrifugation in a Beckman SW40 rotor at 39,000 rpm for 90 minutes at 4°C. For RNase A and EDTA experiments, approximately 20 A_260_ units of S1 was treated with either 10 ng/mL RNase A on ice for 30 minutes or 50 mM EDTA at room temperature for 30 minutes and then loaded onto separate linear 15–60% sucrose gradient (see Supporting Information, Fig. S1 for polyribosome profiles). The polyribosome profile for all gradients was determined by A_254_ with an ISCO gradient fractionator and fractionated into 1-mL fractions by upward displacement. Carrier E. coli RNA (40 µg) and 0.525 ng ‘utility control’ (UT2) mRNA (Universal ScoreCard control poly(A)^+^-RNAs, Amersham Biosciences), which is comprised of intronic yeast sequences, were added to 400 µL of each fraction prior to RNA extraction, as described above. The UT2 mRNA is used to correct for loss of RNA in the extraction procedure [45]. An aliquot of the extracted RNA (10 µL) was reverse transcribed using *Fmr1* and UT2 gene-specific primers. A standard curve was prepared by diluting fraction 6 into 10-fold dilutions and the amount of RNA lost in extraction was determined by quantification of UT2 RNA in each fraction. The relative abundance of each *Fmr1* transcript isoform was determined across the sucrose gradient by qRT-PCR and then normalized with the UT2 data from each fraction. For the EDTA control experiments, fractions 1–5 were combined to represent the ‘top’ of the gradient, while fractions 6–10 were combined to represent the ‘bottom’ of the gradient. *Fmr1* transcript isoforms were quantified in both fraction pools.

### RiboTag Analysis

Specific tagging of neuronal ribosomes with the hemagglutinin epitope (HA) was achieved by crossing the RiboTag mouse [39] with a mouse expressing Cre recombinase from the *Eno2* [40] promoter (Jackson Laboratories). Immunoprecipitation of tagged polyribosomes was performed as described [39] and the associated transcripts were analyzed by qRT-PCR as described above. The transcript isoform profiles were analyzed as described above and in the text.

### Immunohistochemistry on Eno2-Cre:RiboTag Mouse Brain

Immunohistochemistry was performed as described [39]. Antibodies used for immunohistochemistry are as follows: GFAP (Invitrogen, A11122); HA (Covance, A48101L); NeuN (Millipore, MAB377B).

### Data Processing

All raw expression data for *Fmr1* obtained in experiments (except polyribosome experiments) were normalized to the raw values for the housekeeping gene MuP0, measured in each sample, to allow comparison of *Fmr1* data between samples. For polyribosome experiments, raw data obtained for *Fmr1* in each sample was normalized to the introduced UT2 RNA to account for uneven RNA loss during extraction.

## Supporting Information

Figure S1 **Polyribosome profiles of mouse brain in the absence of detergent.**
(PDF)

Figure S2 **Polyribosome association data for the major abundant** ***Fmr1*** **transcript isoforms.**
(PDF)

Figure S3 **Polyribosome association data for the low abundant** ***Fmr1*** **transcript isoforms.**
(PDF)

Figure S4 **Levels of** ***NeuN*** **and** ***GFAP*** **transcripts on neuron-specific ribosomes enriched from adult mouse brain.**
(PDF)

Figure S5 **Schematic representation of alternative splicing of the** ***Fmr1*** **gene and qRT-PCR primers.**
(PDF)

Figure S6 **Agarose gel showing the size of the amplification products of the primer sets that identify the major abundant** ***Fmr1*** **transcripts**
(PDF)

Figure S7 **Agarose gel showing the size of the amplification products of the primer sets that identify the low abundance** ***Fmr1*** **transcripts.**
(PDF)
