# Supplementary material for: Fmr1 Transcript Isoforms: Association with Polyribosomes; Regional and Developmental Expression in Mouse Brain
Source: PLoS One. 2013 Mar 7;8(3):e58296. doi: 10.1371/journal.pone.0058296 (PMC3591412; doi:10.1371/journal.pone.0058296)

Fig. S1. Polyribosome profiles of mouse brain extracted as in the absence of detergent (Materials and Methods).

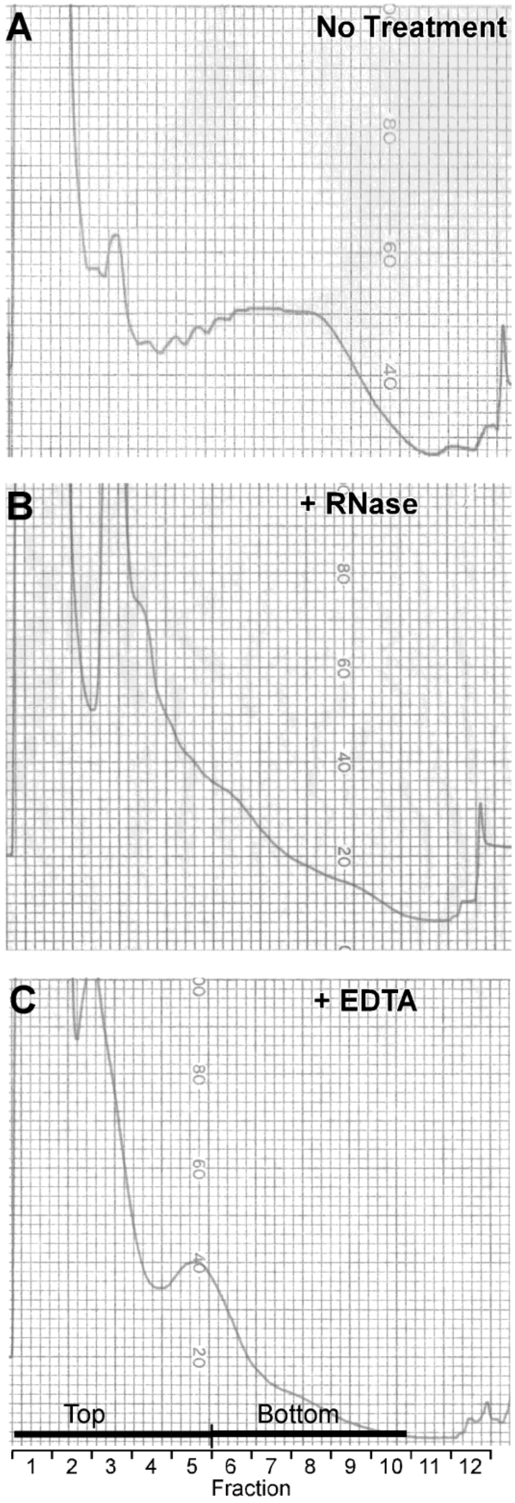

Supplement: Figure S1 — Polyribosome profiles of mouse brain in the absence of detergent. (PDF) [file pone.0058296.s001.pdf]
