# Supplementary figures and images for: Fmr1 Transcript Isoforms: Association with Polyribosomes; Regional and Developmental Expression in Mouse Brain
Source: PLoS One. 2013 Mar 7;8(3):e58296. doi: 10.1371/journal.pone.0058296 (PMC3591412; doi:10.1371/journal.pone.0058296)

Fig. S2. Polyribosome association data for the major abundant *Fmr1* transcript isoforms.

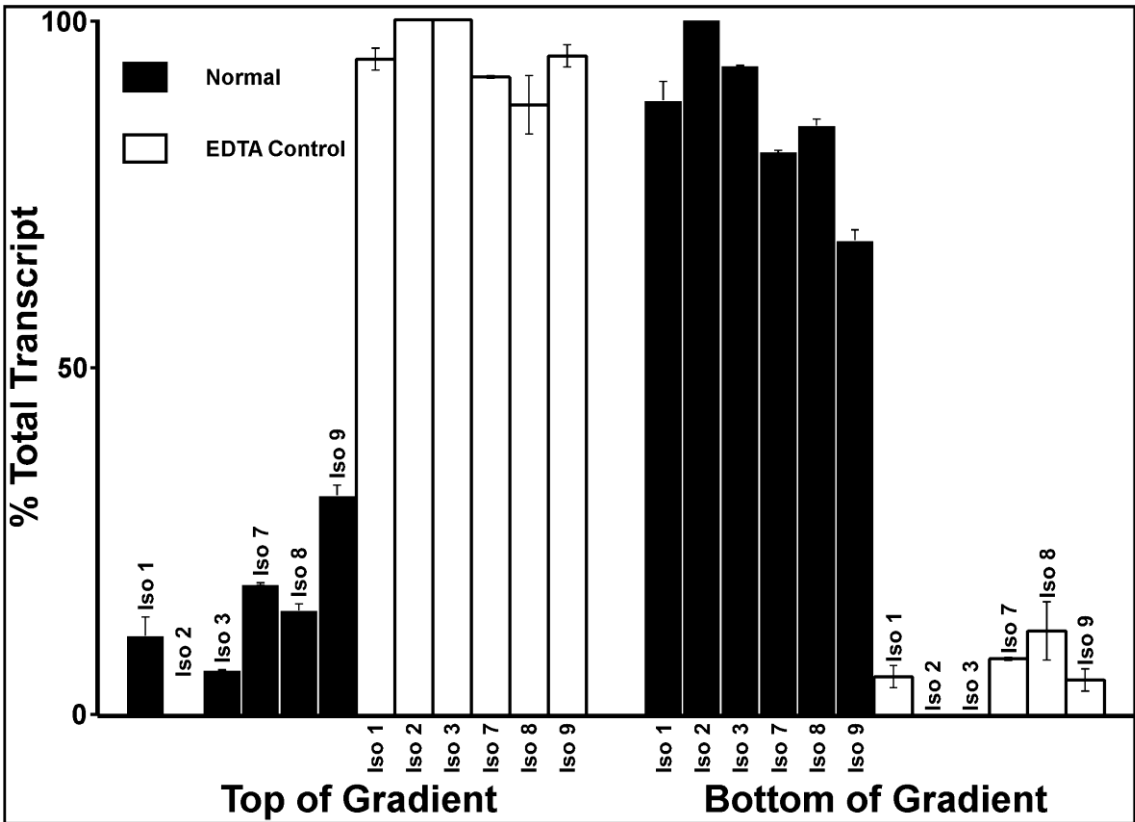

Supplement: Figure S2 — Polyribosome association data for the major abundant Fmr1 transcript isoforms. (PDF) [file pone.0058296.s002.pdf]

Fig. S3. Polyribosome association data for the low abundant *Fmr1* transcript isoforms.

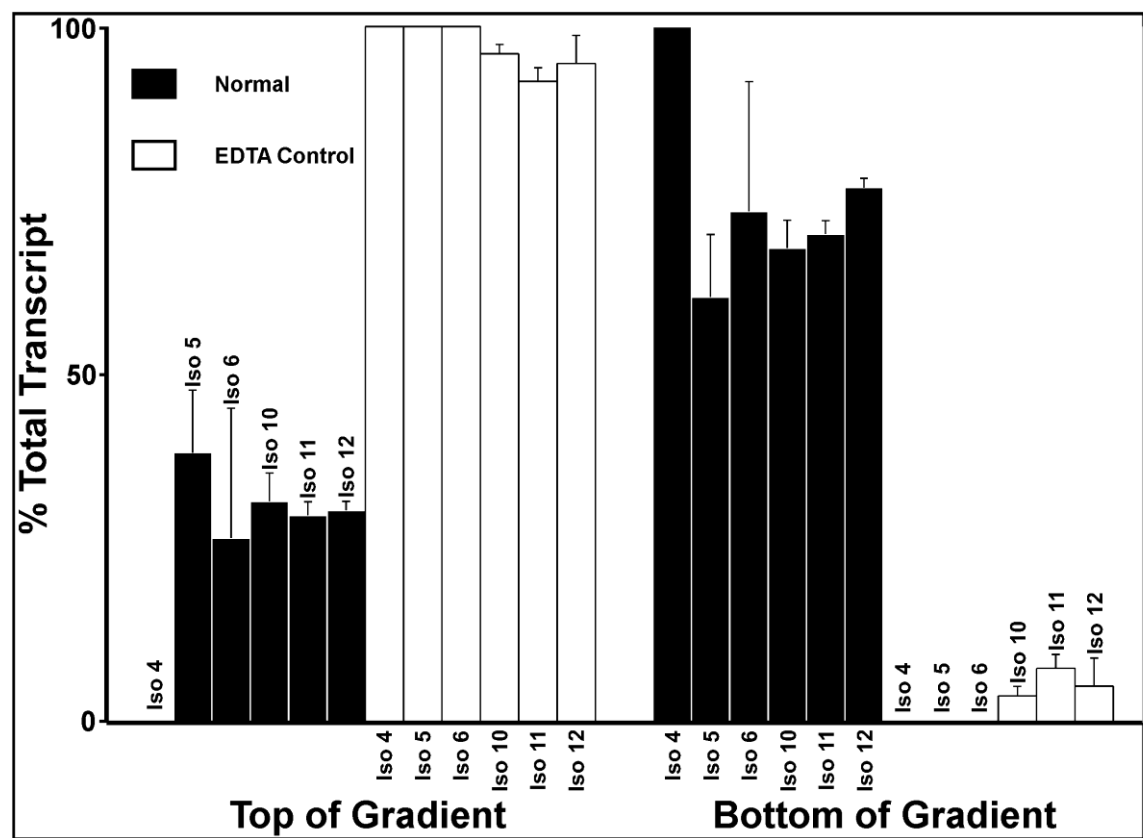

Supplement: Figure S3 — Polyribosome association data for the low abundant Fmr1 transcript isoforms. (PDF) [file pone.0058296.s003.pdf]
