# Supplementary material for: Fmr1 Transcript Isoforms: Association with Polyribosomes; Regional and Developmental Expression in Mouse Brain
Source: PLoS One. 2013 Mar 7;8(3):e58296. doi: 10.1371/journal.pone.0058296 (PMC3591412; doi:10.1371/journal.pone.0058296)

**Fig. S4. Levels of *NeuN* and *GFAP* transcripts on neuron-specific ribosomes enriched from adult mouse brain.**

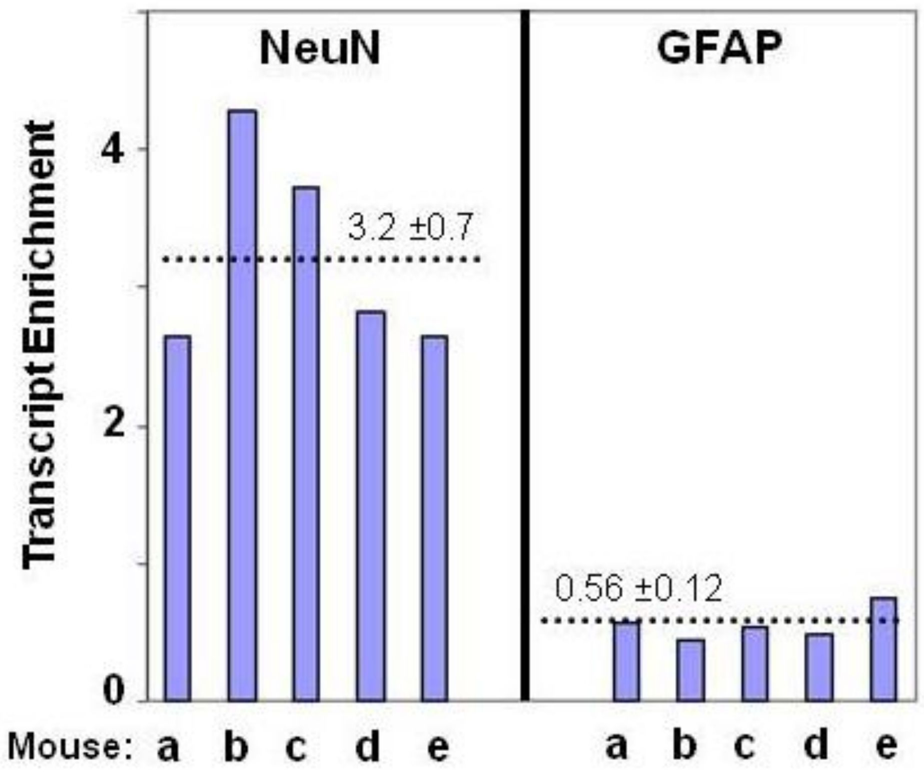

Supplement: Figure S4 — Levels of NeuN and GFAP transcripts on neuron-specific ribosomes enriched from adult mouse brain. (PDF) [file pone.0058296.s004.pdf]
