# Supplementary material for: Fmr1 Transcript Isoforms: Association with Polyribosomes; Regional and Developmental Expression in Mouse Brain
Source: PLoS One. 2013 Mar 7;8(3):e58296. doi: 10.1371/journal.pone.0058296 (PMC3591412; doi:10.1371/journal.pone.0058296)
Supplement: Figure S5 — Schematic representation of alternative splicing of the Fmr1 gene and qRT-PCR primers. (PDF) [file pone.0058296.s005.pdf]

Fig. S5. Schematic representation of alternative splicing of the *Fmr1* gene.

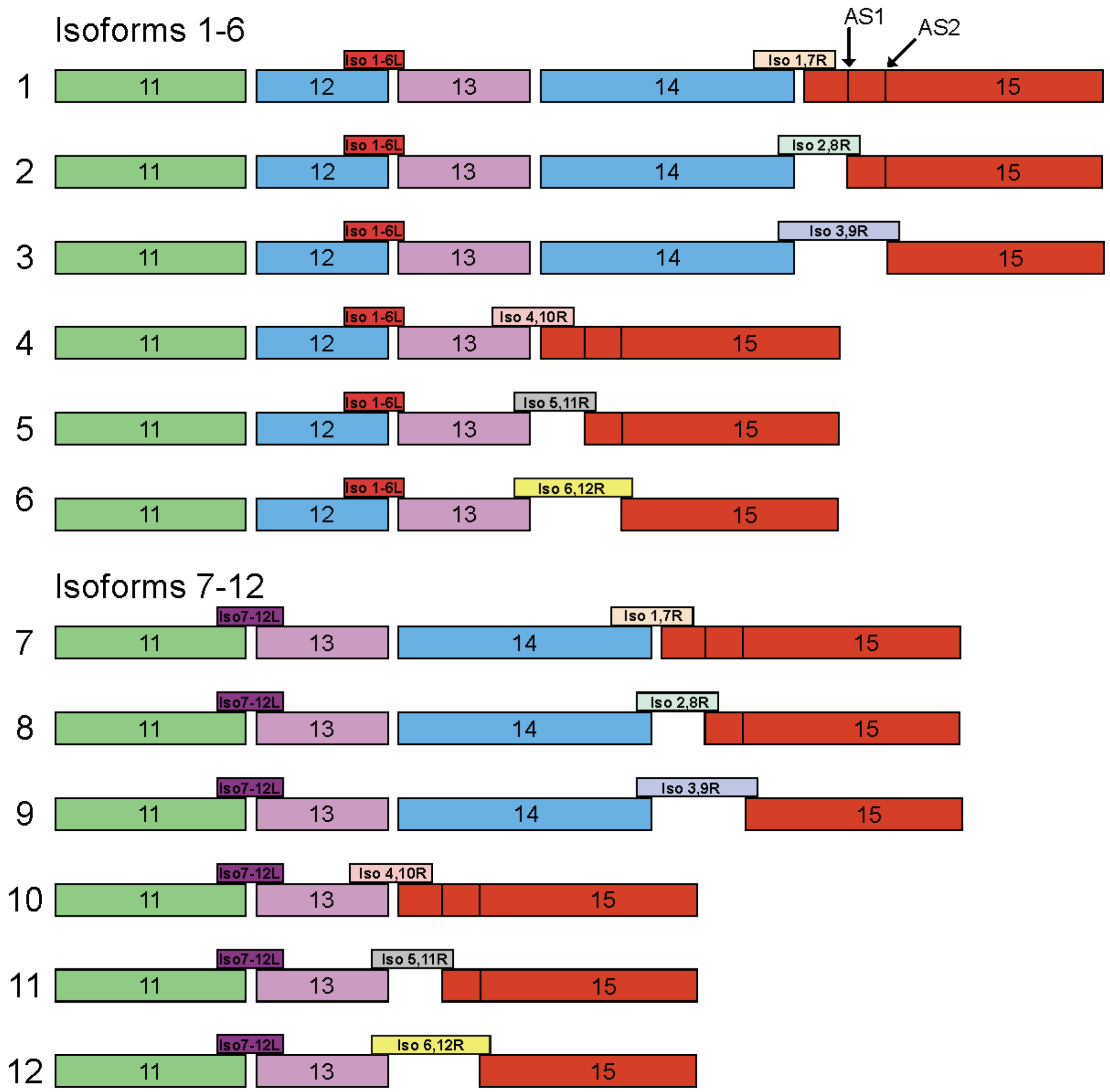

***Fmr1* transcript-specific primers**

Forward

Iso 1-6L: aaggcttggcagggtatg

Iso 7-12L: aaaagtccagaggggtatggta

Reverse

Iso 1, 7R: ctctgtggtcagattctgtttcag

Iso 2, 8R: cagattctgaagtatatccaggac

Iso 3, 9R: cctctgttggagctgaagtatatc

Iso 4, 10R: tgcttcagaattagttcctttaaatag

Iso 5, 11R: tcagattctttaaatagttcaggtg

Iso 6, 12R: tctgttggagctttaaatagttcag
