# Supplementary material for: Fmr1 Transcript Isoforms: Association with Polyribosomes; Regional and Developmental Expression in Mouse Brain
Source: PLoS One. 2013 Mar 7;8(3):e58296. doi: 10.1371/journal.pone.0058296 (PMC3591412; doi:10.1371/journal.pone.0058296)

Fig. S7. Agarose gel showing the size of the amplification products of the primer sets that identify the low abundance *Fmr1* transcripts.

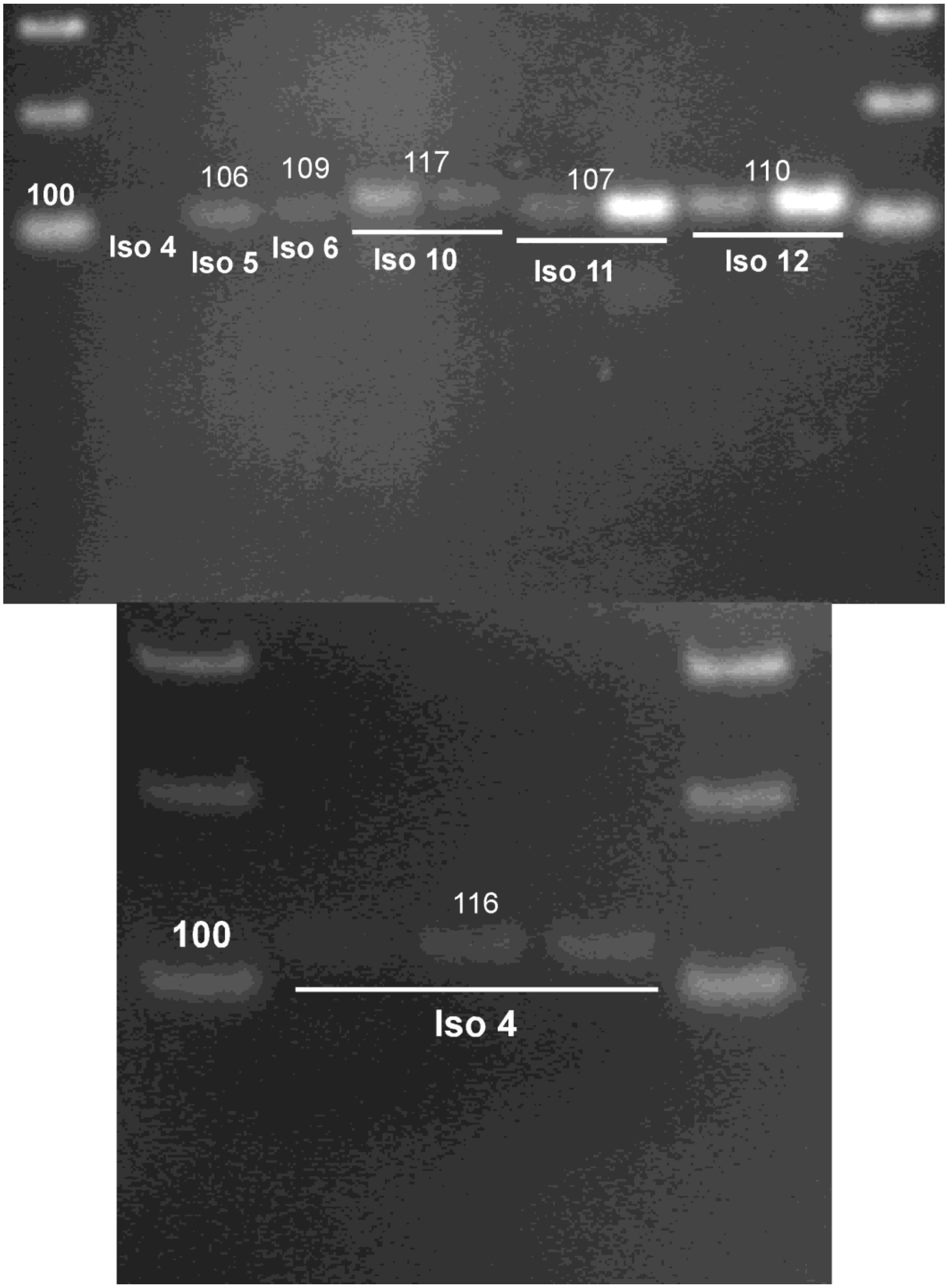

Supplement: Figure S7 — Agarose gel showing the size of the amplification products of the primer sets that identify the low abundance Fmr1 transcripts. (PDF) [file pone.0058296.s007.pdf]
